# A Case of Mixed Type II Cryoglobulinemic Vasculitis Associated With Marginal Zone B-cell Lymphoma

**DOI:** 10.7759/cureus.33001

**Published:** 2022-12-27

**Authors:** Larabe Farrukh, Lisa Rosenberg, Hafiza H Waqar, Javaria Tehzeeb, Muhammad F Akhtar, Pooja Chaukiyal

**Affiliations:** 1 Internal Medicine, Albany Medical Center, Albany, USA; 2 Oncology Hematology, Albany Medical Center, Albany, USA

**Keywords:** rituximab therapy, non-hodgkins lymphoma, mixed cryoglobulinemia, cryoglobulins, small vessel vasculitis

## Abstract

Our patient is a male in his 40s with a past medical history of sickle cell trait, factor V Leiden mutation, marginal zone B-cell lymphoma, gastric mucosa-associated lymphoid tissue (MALT) lymphoma, and sarcoidosis who presented with the complaint of hemoptysis, dyspnea, abdominal pain, arthralgias, peripheral edema of the lower extremities with petechial rash, and oliguria.

Investigations revealed acute kidney injury and bilateral transudative pleural effusion. Serology was positive for elevated rheumatoid factor, low complement components, and cryoglobulins. Renal biopsy showed membranoproliferative cryoglobulinemic glomerulonephritis with deposition of monoclonal IgM and IgG3 with kappa light chain and C3 component. The patient was diagnosed with mixed type II cryoglobulinemic vasculitis in the setting of untreated marginal B-cell lymphoma. He had a complex clinical course, requiring multiple intubations, hemodialysis, and treatment with intravenous immunoglobulin, plasmapheresis, steroids, and chemotherapy, to which he initially responded. During treatment, he developed cardiomyopathy associated with congestive heart failure and passed away due to cardiac arrest.

We present a rare case of mixed type II cryoglobulinemic vasculitis secondary to untreated marginal zone B-cell lymphoma in a hepatitis C virus (HCV) negative patient, which has not been reported before.

## Introduction

Cryoglobulinemia can be classified into three types: type I, which is composed of a single monoclonal immunoglobulin; type II, which is characterized by immune complexes composed of polyclonal IgG and monoclonal IgM; and lastly, type III, which is composed of immune complexes containing polyclonal IgG and polyclonal IgM. Type I is mainly found in patients with lymphoid disorders such as lymphomas, Waldenstrom's macroglobulinemia, and multiple myeloma. Mixed cryoglobulinemia (MC) type II and III can be associated with various infections, particularly hepatitis C virus (HCV), and rarely with autoimmune diseases.

## Case presentation

Our patient is a 49-year-old male with a past medical history of sickle cell trait, factor V Leiden mutation, marginal zone B-cell lymphoma, gastric mucosa-associated lymphoid tissue (MALT) lymphoma, and sarcoidosis who presented with the complaint of cough with hemoptysis, dyspnea, abdominal pain, arthralgias, peripheral edema, petechiae on lower extremities and oliguria. 

In terms of past medical history, the patient was diagnosed with marginal B-cell lymphoma in 2018 (bone marrow biopsy proven). In 2019, he had an endoscopy done with a biopsy, which was negative for *Helicobacter pylori (H. pylori)* but positive for MALT lymphoma involving the stomach and duodenal mucosa. He was also found to have prominent splenomegaly. Treatment was not initiated as the patient still had early disease, but he followed up with his hematologist on a regular basis. He had hilar lymphadenopathy on a previous chest X-ray done in 2018, which was followed by bronchoscopy with bronchoalveolar lavage (BAL), and a biopsy positive for non-caseating granulomas, suggestive of sarcoidosis. He did not receive treatment for sarcoidosis due to the absence of active symptoms.

According to the patient, his joint pain started after a mechanical fall while playing soccer. In the following days, he developed a worsening cough with hemoptysis, nasal congestion, and pleuritic chest pain. He took non-steroidal anti-inflammatory drugs (NSAIDs) for arthralgias and developed decreased urinary output despite adequate water intake.

Upon arrival at the hospital, the patient was found to be hypoxic and was placed on nasal cannula oxygen. CT chest showed diffuse bilateral patchy consolidations, moderate-sized pleural effusions, and bilateral hilar lymphadenopathy, which had increased from his last positron emission tomography (PET) scan. Thoracentesis removed 1 L of fluid from the right lung, which was transudative in nature.

**Figure 1:**
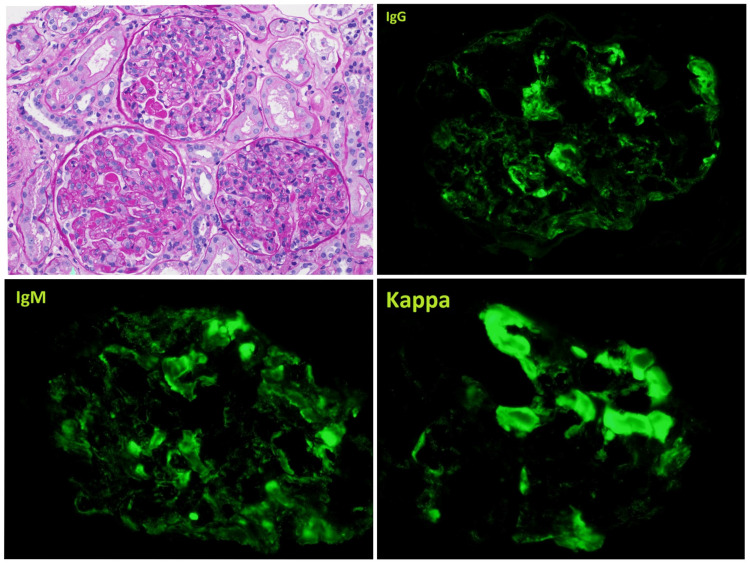
Diffuse proliferative cryoglobulinemic glomerulonephritis, type II. Immunofluorescence microscopy shows monoclonal deposition of both IgM and IgG3 with kappa light chain

Labs showed hemoglobin (Hb) 11.4 (13.6-16.7 g/dL), WBC count 11.9 (4.0-9.0 10*3/uL), platelet count 195 (130-350 10*3/uL), elevated serum creatinine 1.98 (0.8-1.4 mg/dL), and urine microscopy revealed muddy brown casts. Erythrocyte sedimentation rate (ESR) 31 (0-15mm/hr) and C-reactive protein (CRP) 18 (<8.0 mg/L) were elevated. Serology showed an elevated rheumatoid factor of 240 (normal <14 IU/ml). Complement components, including C3 77.5 (87-200 mg/dL), C4 <8 (19-52 mg/dL), C1q component 7.6 (10.3 - 20.3 mg/dL), and C1q binding protein < 1.2 (normal 10.2-20.3 μg/ml) were low. Cryoglobulins were positive. Other serologies, including anti-RNP antibodies (Ab), antinuclear antibodies (ANA), anti-double stranded DNA (anti-dsDNA) Ab, anti-smith Ab, anti-SSA/SSB, and early Sjögren's panel, were negative. Aantineutrophil cytoplasmic antibodies (ANCA) titers (c-ANCA, p-ANCA, atypical ANCA, anti-proteinase-3 (anti-PR3) Ab, anti-myeloperoxidase (anti-MPO) Ab) were within normal limits. Antiphospholipid panel was normal. Renal biopsy showed membranoproliferative cryoglobulinemic glomerulonephritis with deposition of monoclonal IgM and IgG3 with kappa light chain and C3 component deposition (Figure 1).

Considering the patient's past medical history of sarcoidosis, we were concerned about IgA nephropathy; however, serum IgA levels were normal, and renal biopsy was negative for IgA deposition. ANCA-associated vasculitis, including granulomatosis with polyangiitis (GPS), eosinophilic granulomatosis with polyangiitis (EGPA), and microscopic polyangiitis (MPA), were ruled out with normal ANCA titers, and renal biopsy did not show pauci-immune crescentic glomerulonephritis which is specific for ANCA vasculitis. The patient had elevated rheumatoid factor (RF) and very low complement levels. This was supplemented by renal biopsy findings of membranoproliferative cryoglobulinemic glomerulonephritis with monoclonal deposition of IgM and IgG3, leaning us towards a diagnosis of mixed type II cryoglobulinemic vasculitis [1]. Mixed type II cryoglobulinemia can be associated with autoimmune disorders like mixed connective tissue disease (MCTD), systemic lupus erythematosus (SLE), and Sjögren's syndrome. However, the patient had no positive review of the systems, and serology was negative for these disorders. As type II and III cryoglobulinemia are commonly found in persistent hepatitis infection, this was ruled out by a negative hepatitis B/C viral panel.

Plasmapheresis was initiated with rituximab directed against marginal B-cell lymphoma, later changed to rituximab, cyclophosphamide, vincristine, and prednisone (R-CVP) in the setting of worsening hemoptysis due to diffuse alveolar hemorrhage. Other therapies included intravenous immunoglobulins, steroids, diuretics, and hemodialysis. Rasburicase was given for possible tumor lysis syndrome in the setting of elevated uric acid and lactate dehydrogenase (LDH). Anakinra was added due to concerns for macrophage activation syndrome in the setting of pancytopenia, hypofibrinogenemia, elevated D-dimers, and markedly elevated ferritin. Despite these treatments, the patient had a complex hospital course, requiring multiple sessions of hemodialysis for acute renal failure and intubation for acute hypoxic respiratory failure secondary. He was eventually weaned off mechanical ventilation and improved over the course of a few weeks. 

The patient was discharged on oral steroids (prednisone 40 mg daily), outpatient plasmapheresis (two times per week), and chemotherapy (R-CVP). He continued to follow up with hematology. Unfortunately, he presented a few weeks later to an outside hospital with worsening dyspnea and altered mental status. He passed away shortly after arrival due to acute respiratory failure and cardiac arrest (pulseless electrical activity) despite receiving life-saving measures.

## Discussion

The etiopathogenesis of mixed cryoglobulinemic (MC) vasculitis is not completely understood as it remains a rare diagnosis. Hepatitis C virus (HCV) infection is suggested to play a causative role in some cases; however, in countries where the prevalence of HCV is extremely low, most cases of MC were associated with underlying lymphoproliferative and autoimmune diseases [2,3]. Currently, there is there no established diagnostic criteria for MC. However, in 1989 the Italian Group for the Study of Cryoglobulinemias proposed a preliminary criterion for MC classification (Table 1). As per this criterion, the detection of circulating mixed cryoglobulins, low C4 levels, and orthostatic skin purpura are the hallmark features of this disease [4,5].

**Table 1:** Proposed criteria for the classification of patients with mixed cryoglobulinemia

Criteria	Present in our case
Major criteria	
Mixed cryoglobulins (serologic)	Yes
Low C4 levels (serologic)	Yes
Leukocytoclastic vasculitis (pathologic)	Skin biopsy not performed
Purpura (clinical)	Yes
Minor criteria	
Rheumatoid factor (serologic)	Yes
Hepatitis C positive (serologic)	No
Hepatitis B positive (serologic)	No
Clonal B-cell infiltrates - bone marrow (pathologic)	Yes
Chronic hepatitis (clinical)	No
Membranoproliferative glomerulonephritis (clinical)	Yes
Peripheral neuropathy (clinical)	No
Skin ulcers (clinical)	No

While planning treatment for MC, it is important to treat any underlying disorder like an untreated lymphoproliferative disease, as in this case. Patients with severe or life-threatening manifestations require very close monitoring and urgent immunosuppressive therapy (high-dose corticosteroids, cyclophosphamide, rituximab, and plasmapheresis). Rituximab plays a major role in the current approach of MC management. The largest data on the use of rituximab in MC comes from a multicenter survey in which the use of rituximab in combination with corticosteroids was found to achieve the greatest benefit in terms of clinical response [6-8]. 

## Conclusions

Marginal zone lymphoma presenting as mixed cryoglobulinemic vasculitis in an HCV-negative patient, with the presence of two monoclonal immunoglobulins, including IgM with RF activity and IgG3, is of rare occurrence and has not been previously reported. HCV-negative MC is clinically comparable to HCV-associated disease; however, it usually presents with a worse clinical course.
